# An artificial neural network combined with response surface methodology approach for modelling and optimization of the electro-coagulation for cationic dye

**DOI:** 10.1016/j.heliyon.2022.e08749

**Published:** 2022-01-12

**Authors:** Manisha S. Kothari, Kinjal G. Vegad, Kosha A. Shah, Ashraf Aly Hassan

**Affiliations:** a Civil and Environmental Engineering Department, National Water and Energy Center, United Arab Emirates University, Al-Ain, Abu-Dhabi, United Arab Emirates; b Civil Engineering Departments, Faculty of Technology and Engineering, The Maharaja Sayajirao University of Baroda, Vadodara, 390001, India

**Keywords:** Electrocoagulation process, Response surface methodology, Artificial neural network, Colour removal efficiency, Electrical energy consumption

## Abstract

An artificial neural network (ANN) approach with response surface methodology (RSM) technique has been applied to model and optimize the removal process of Brilliant Green dye by batch electrocoagulation process. A multilayer perceptron (MLP) - ANN model has been trained by four input neurons which represent the reaction time, current density, pH, NaCl concentration, and two output neurons representing the dye removal efficiency (%) and electrical energy consumption (kWh/kg). The optimized hidden layer neurons were obtained based on a minimum mean squared error. The batch electrocoagulation process was optimized using central composite design with RSM once the ANN network was trained and primed to anticipate the output. At optimized condition (electrolysis time 10 min, current density 80 A/m^2^, initial pH 5 and electrolyte NaCl concentration 0.5 g/L), RSM projected decolorization of 98.83% and electrical energy consumption of 14.99 kWh/kg. This study shows that the removal of brilliant green dye can be successfully carried out by a batch electrocoagulation process. Therefore, the process is successfully trained by ANN and optimized by RSM for similar applications.

## Graphical abstract

**Figure:**
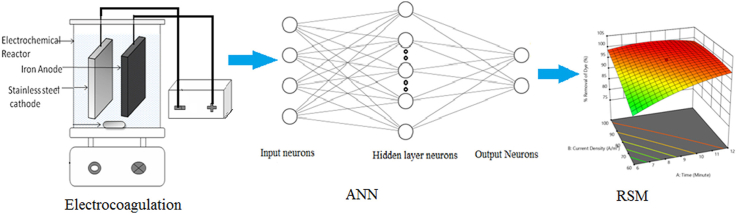

## 1 Introduction

Industries like textiles, leather, paper, pulp, printing, and dyeing are major consumers of synthetic dyes. Such industries produce large quantities of colored wastewater which cause significant adverse effects on the environment. In receiving water bodies, the dyes present in wastewater cause unesthetic appearance, prevention of sunlight penetration resulting in underdeveloped aquatic plant growth and toxicity to aquatic life [1, 2]. It is, therefore, necessary to treat the wastewater, particularly to decolorize the wastewater before discharging it into the surface water bodies. Various techniques have been practiced for dye removal, i.e., adsorption [3], chemical coagulation [4], membrane processes [5], biodegradation [6], and advanced oxidation processes [7]. Coagulation, adsorption, and membrane processes produce concentrated dye sludge posing a disposal problem. Membranes are known for their problem of “flux decline” [8, 9]. Biodegradation processes require a favourable environment, free from toxicity [10]. Advanced oxidation processes such as UV photolysis, electrochemical oxidation, Fenton processes, and their combinations suffer from the limitations of high cost, high energy consumption, and secondary pollutant generation [11]. The electrocoagulation process overcomes the limitations of these existing treatment processes [12]. Processes like adsorption, although competitive with electrocoagulation, needs an adsorbent (mostly activated carbon) adding to the cost of treatment. Moreover, activated carbon adsorption process results in the formation of a large amount of sludge. Because of its cost-effectiveness and simplicity, the electrocoagulation procedure has received much interest. The method eliminates the addition of chemicals and reduces the sludge generation [13].

The electrocoagulation process involves the in-situ generation of coagulants by metal ions produced from electro-dissolution of a sacrificial anode [14]. The metal ions formed cause the flocculation and sedimentation of pollutants. Cathodic action also aids in the removal of pollutants by the formation of hydrogen gas, causing floatation or by deposition of the pollutant on the cathode surface [15]. Usually, Fe and Al are utilized as electrodes. Various contaminant species have been removed using electrocoagulation are: sulfide [16], phosphates [17], fluoride [18], and dyes such as Acid Red 14 [19], Reactive Black-5 [20].

The majority of articles in the literature focus solely on the pollutant's removal efficiency. On the contrary, as the electrocoagulation process is a current induced approach, the removal should be optimized with the energy consumption to achieve cost reduction for industrial application. An artificial neural network (ANN)- response surface methodology (RSM) combination is an emerging reproducible process modelling and optimization technique used by a few researchers [21, 22] for dye removal, to save time and cost of experimentation. For future comparable applications, the process must be simulated and optimized. The current study successfully integrates two essential components, colour removal efficiency (%) and electrical energy consumption, to illustrate the efficiency and cost-effectiveness of the electrocoagulation method. In addition, an ANN is used to represent the process. The data from the ANN model is then used to optimise the process using the RSM.

The electrocoagulation process works by electro-oxidation of Fe anode, which produces Fe(OH)_n_, (here the value of n could be 2 or 3) as explained in Eqs. (1), (2), (3), and (4) [23].

At anode:

(1) $$4Fe_{s}\to 4Fe_{\left(aq\right)\;}^{+2}+8e^{-}$$

(2) $$4Fe_{\left(aq\right)}^{+2}+10H_{2}O_{\left(l\right)}+O_{2}\to 4Fe{{\left(OH\right)}_{3}}_{\left(s\right)}+8H_{\left(aq\right)}^{+}$$

At cathode:

(3) $$8{\text{H}}_{\left(\text{aq}\right)}^{+}+8{\text{e}}^{-}\to 4{\text{H}}_{2\left(\text{g}\right)}$$

Inclusive Reaction:

(4) $$4Fe_{\left(s\right)}+10H_{2}O_{\left(l\right)}+O_{2\left(g\right)}\to 4Fe{{\left(OH\right)}_{3}}_{\left(s\right)}+4H_{2\left(g\right)}$$

With subsequent coagulation and settling, the Fe(OH)_n(s)_ forms a complex with the pollutant or adsorbs it. The metal oxides produced by the anode material form hydroxide - metallic complexes i.e., Fe_2_(OH)_2_^4+^ and Fe_2_(OH)_4_^5+^ which are responsible for complications of the pollutants [24]. Other complexes involved in the adsorption of pollutants including Fe(OH)_2_^+^, Fe(OH)^2+^, Fe(OH)_4_^-^, Fe(H_2_O)_4_(OH)_2_^+^, Fe(H_2_O)_5_(OH)^2+^, and Fe_2_(H_2_O)_6_(OH)_4_^4+^ [25].

An electrocoagulation technique was used to treat a synthetic Brilliant Green (BG) dye solution in this investigation. The influence of four factors on the process was investigated: time, current density, pH, and concentration of the supporting electrolyte (NaCl). This study optimized two essential responses: decolorization efficiency and electrical energy usage. The data from preliminary experiments were added to an ANN and the stimulated data from the trained ANN was optimized using RSM.

An ANN mechanism is based on simulation with a human brain. i.e., ANNs are stimulated by biological neural systems. A computational model of natural neurons is called an artificial neuron. Each ANN is made up of artificial neurons that are grouped into layers and connected in a parallel manner. An ANN network is made up of inputs that are multiplied by weights to keep the intensity of the signal's constant, and then computed by a mathematical function that defines the neuron's activity. Weights are assigned to the signals of each neuron during an ANN training. For this study, the backpropagation method is used. Backpropagation is a learning mechanism which utilizes a gradient descent method while training the network. The training of a backpropagation algorithm works in the following steps: input forward feeding followed by error estimation, backpropagation, and adjustment of weights to the input variables. The RSM is an optimization and design technique based on statistics with two sets of variables named as independent and dependent. The relationship between these two sets of variables is analyzed and presented by RSM. Among the various classes of RSM, central composite design (CCD) is the most appropriate model for linear as well as non-linear relations amongst the independent and dependent variable/response. Moreover, in this study, central composite design was used instead of the Box- Behnken design because the ANN responses were available by modelling, and they readily provided the additional data to feed for axial and centre points in the CCD. The CCD being a robust technique, is successfully used recently for optimization by researchers for adsorption of heavy metals on multi-wall carbon nanotubes [26, 27], graphene oxide [28, 29], and oxidative removal of dye by Fenton's reagent [30].

## 2 Material and methodology

### 2.1 Materials and experimental set-up

**Figure 1:**
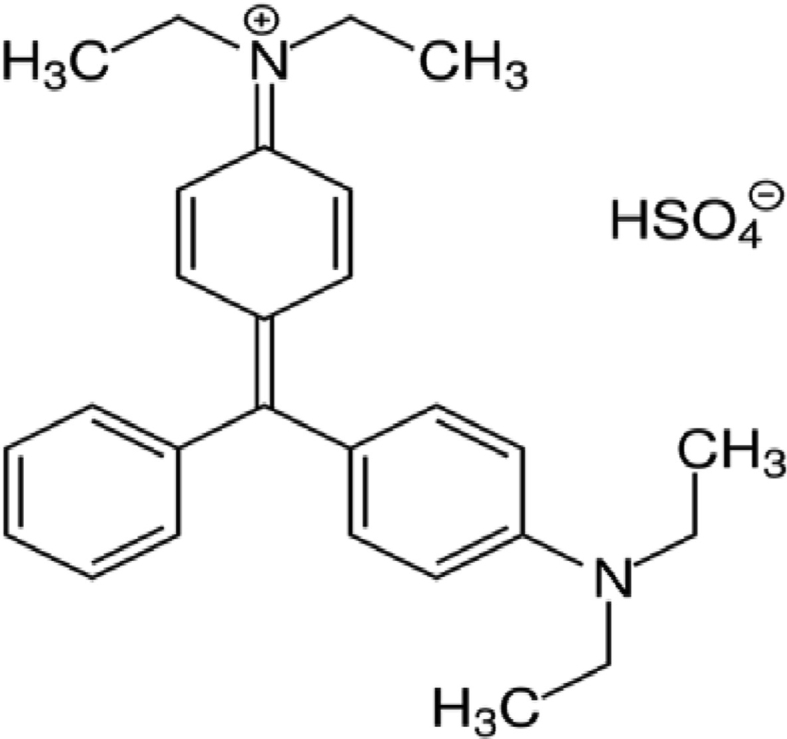
Structure of Brilliant Green dye.

**Figure 2:**
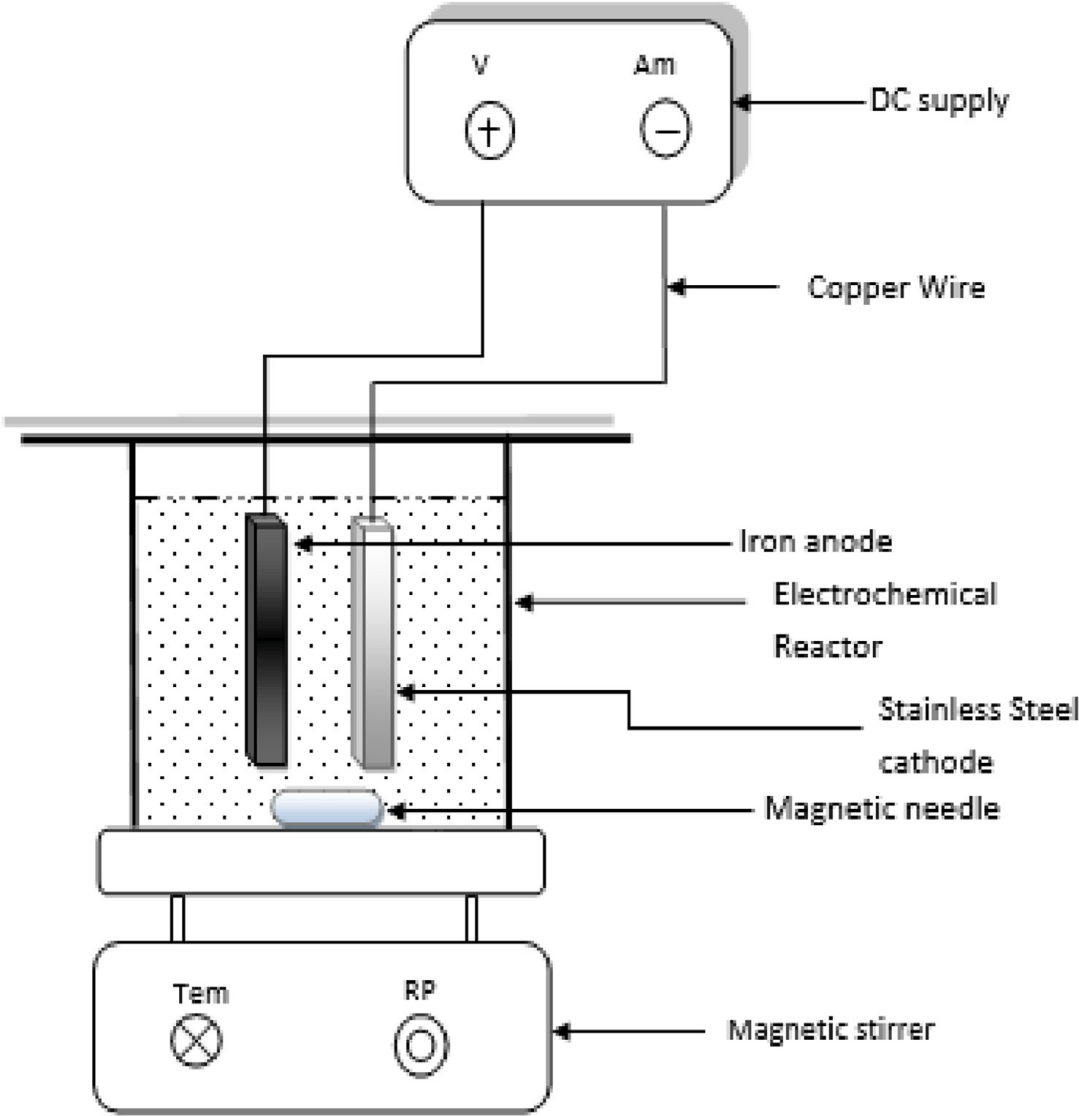
Experimental reactor set-up.

BG dye (Glaxe laboratories ltd. India, 95% purity) was used as the target pollutant. BG dye is an organic hydrogen sulfate salt. The BG dye's distinctive schematic structure is represented in Figure 1 and Table 1 shows its general characteristics. The reactor setup is represented in Figure 2. A polyacrylic reactor of 500 ml capacity consists of one pair of Iron (Fe) as anode and stainless steel as cathode, both with the size of 5.5 cm × 5.0 cm x 0.3 cm. At 1.5 cm inter-electrode spacing, both the anode and the cathode are parallel to each other. A DC power supply of 32V, magnetic stirrer (Janki impax, India), and pH meter (Toshcon, India) were used during the experiments.

**Table 1:** General characteristics of Brilliant Green dye.

Chemical formula	C_27_H_33_N_2_.HO_4_S
λmax	626 nm
Molar mass	482.64 g/mol
Index No.	42040

### 2.2 Batch experimental process and analytical techniques

BG dye was dissolved in distilled water to make a 10 g/L synthetic dye stock solution. Stock solution (2.5 ml) was diluted to 500 ml to obtain 50 mg/L concentrated solution. All the experiments have been carried out at a constant temperature of 30 °C. Synthetic wastewater containing dye (500 ml) was added to the reactor for each batch experiment, and the reactor was powered by a DC power source. All preliminary experiments were carried out by varying one parameter at a time for the following parameters: current density, pH, and the concentration of the supporting electrolyte (NaCl). The samples were collected at constant time intervals and left to settle for 30 min and filtered by Whatman filter paper no 1. The absorbance of BG dye was measured by spectrophotometer (Shimadzu UV-1800). The dye removal efficiency in percentage (%) is obtained by Eq. (5). Where, Abs_0_ is the solution's initial absorbance and Abs_t_ is the solution's ultimate absorbance after treatment at time (t).

(5) $$\mathrm{Color}\;\mathrm{removal}\left(\%\right)=\left[\frac{{\text{Abs}}_{0}–{\text{Abs}}_{\text{t}}}{{\text{Abs}}_{0}}\right]\times 100$$

Electrical energy consumption is calculated according to Eq. (6) and Eq. (7); where, electrical energy (KWh) is denoted by E_c,_ applied voltage (Volt) is denoted by U, I is the current (Ampere), t_EC_ is reaction time (hours), C₀ is initial dye concentration (mgL^−1^), R_dye_ is the dye removal efficiency, V is the volume of sample that has been treated.

(6) $$E_{c}\;=\;UIt_{EC}$$

(7) $$E_{c}\left(\frac{kWh}{kgdye}\right)=\frac{U\times I\times t_{EC}\times \;1000}{\left(V\times \left(C_{0}\times R_{dye}\right)\right)}\;$$

### 2.3 Artificial neural network modelling

A multilayer perceptron (MLP) is a feed-forward type ANN made up of layers of neurons used in this work. The independent variable inputs are represented by the first layer of neurons. It is possible to determine the relative effect of each input neuron and its intricate interconnections on the observed outcome. The MLP in this work has four input neurons that indicate response time, current density, pH, and NaCl concentration, as well as a single hidden layer of neurons and two output neurons that represent dye removal efficiency (%) and electrical energy consumption. Hundred numbers of data sets were fed, where 70% of the data sets will be employed for training and the remaining 30% for testing and validation. The trial and error method was utilized to determine the digit of neurons required in the hidden layer, with the goal of minimizing the divergence between forecasts and experimental findings. In the present study, TANSIG and PURELIN activation functions were utilized from the input to the hidden layer mapping and from the hidden layer to the output layer mapping, respectively.

### 2.4 Response surface methodology

The impact of four primary influencing parameters: electrolysis duration, current density, pH, and electrolyte concentration, on two responses, colour removal efficiency and electrical energy consumption was estimated by means of second order central composite design. Table 2 shows the input parameter ranges that were determined based on preliminary experimental work. There were five levels for each parameter: the center point, two axial points (±α), two factorial points (±1). The responses were supplied from the created ANN model's output.

**Table 2:** Range and levels of independent parameters.

Independent Variables	Range and levels				
	-α	-1	0	1	+α
Electrolysis Time (x_1,_ minutes)	3	6	9	12	15
Current Density (x_2_, A/m^2^)	40	60	80	100	120
pH (x_3_)	2	4	6	8	10
Electrolyte concentration (NaCl) (x_4_, g/L)	0.2	0.3	0.4	0.5	0.6

The second order model for response variables is represented by Eq. (8) [31].

(8) $$\text{y}={\text{β}}_{0}+\sum_{\text{i}=1}^{\text{k}}{\text{β}}_{\text{i}}{\text{x}}_{\text{i}}+\sum_{\text{i}=1}^{\text{k}}{\text{β}}_{\text{ii}}{\text{x}}_{\text{i}}^{2}+\sum_{\text{i}-1}^{\text{k}-1}\times \sum_{\text{j}=1}^{\text{k}}{\text{β}}_{\text{ij}}{\text{x}}_{\text{i}}{\text{x}}_{\text{j}}+\text{ε}$$

The response variable is indicated by y. β_0,_ β_i_, β_ii,_ β_ij_ are constant coefficient, linear coefficient, second order coefficient, and interaction coefficient, respectively. k is the numbers of independent variables, x_i,_ and x_j_ symbolize the coded values of the parameters, and n represents the number of parameters.

## 3 Results and discussions

### 3.1 ANN modelling

**Figure 3:**
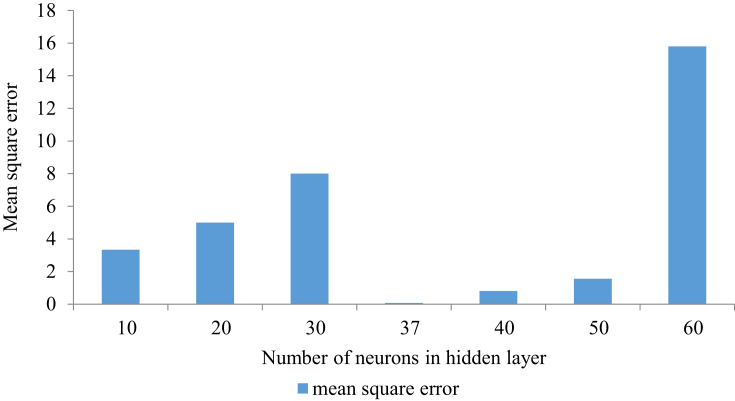
Mean square error Vs numbers of hidden layer neurons.

**Figure 4:**
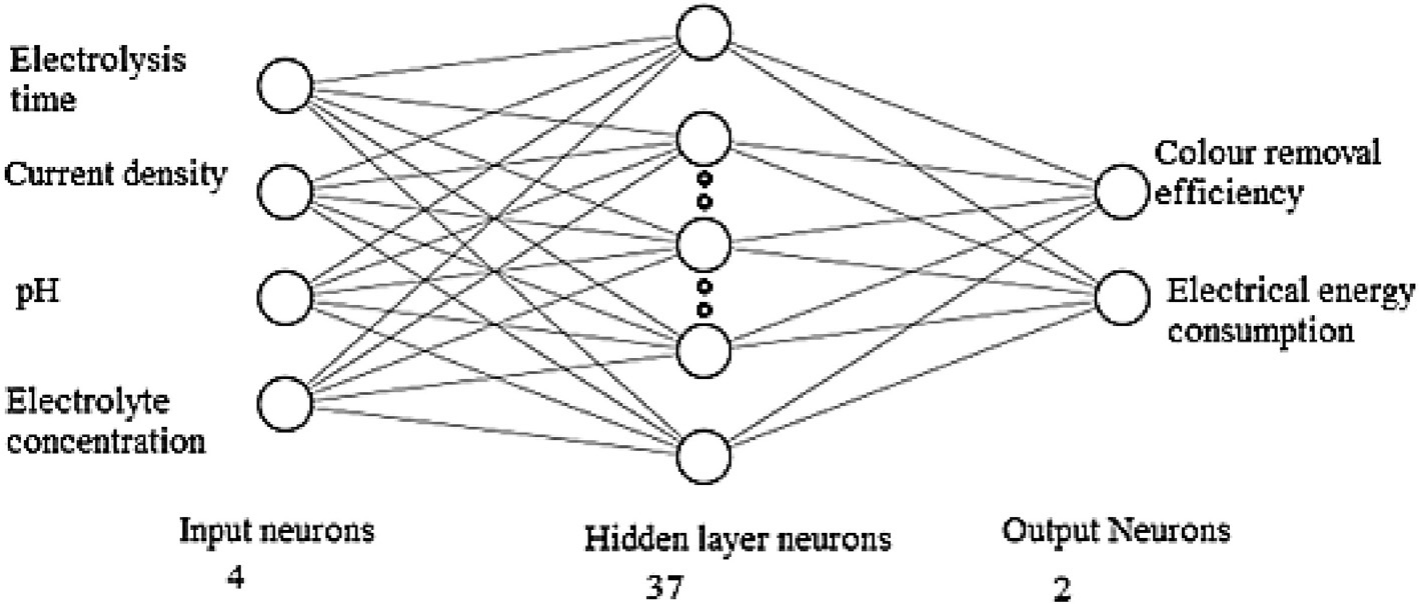
ANN structure.

**Figure 5:**
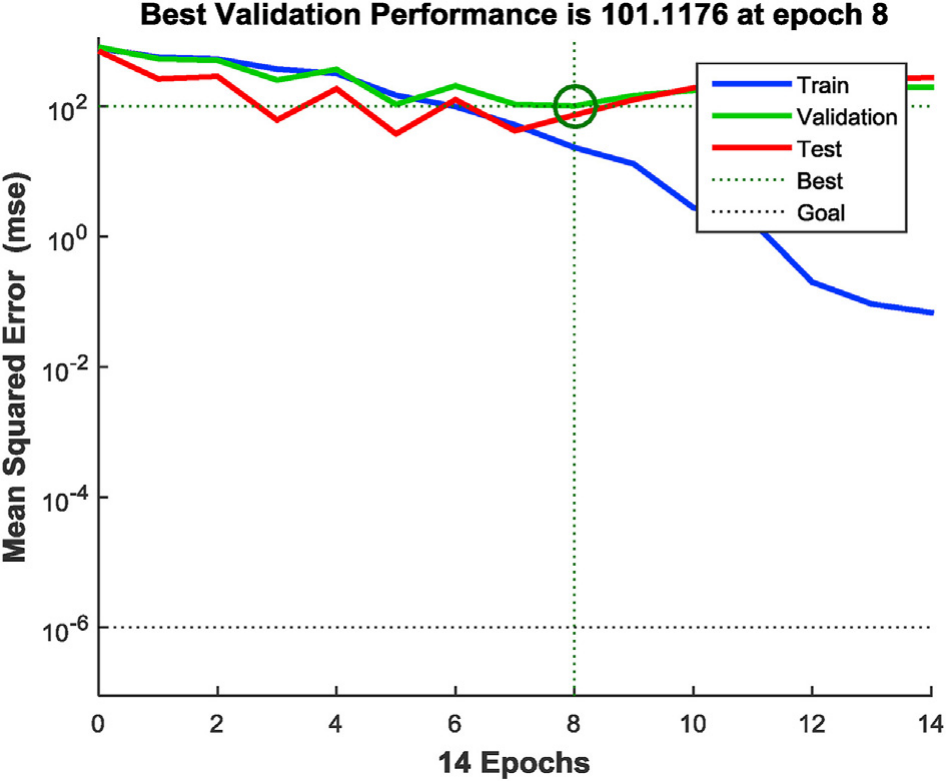
Validation performance plot.

**Figure 6:**
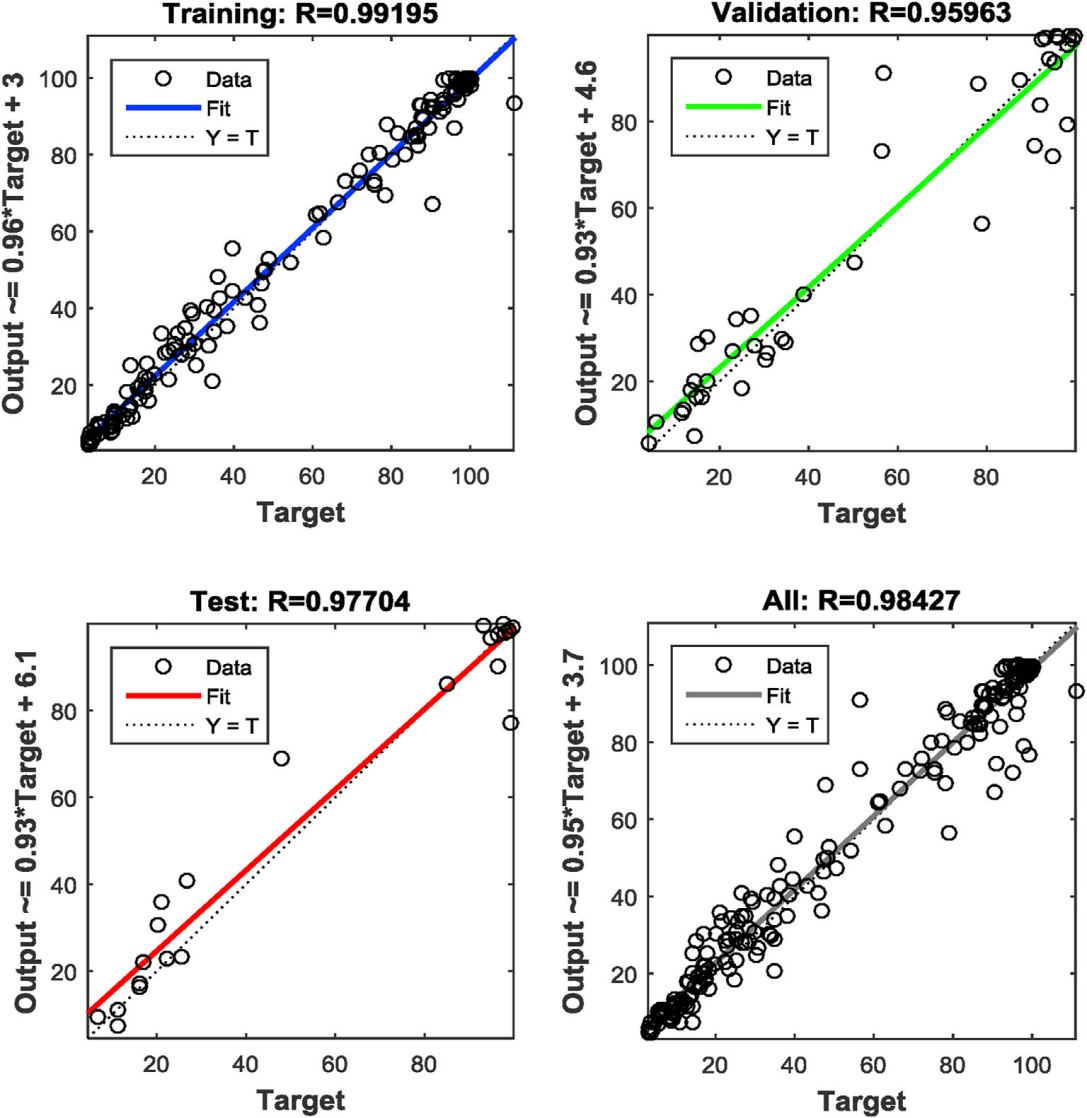
Regression fit of the trained network.

A backpropagation algorithm was used, which trains itself with a large set of input and output data fed into the ANN and computes the mean squared error [32]. The algorithm was initiated with random weight assignment, until the minimum error was observed. Corresponding optimum hidden layer neurons were found out. The training was carried out for 2,000 iterations. The ANN was tested for hidden layer neurons between 10 and 60. Figure 3 shows the mean square error vs. hidden layer neurons. From Figure 3, the optimized hidden layer neurons were found to be 37 corresponding to the minimum mean square error of 0.068. A representation of the MLP diagram for an ANN structure can be observed in Figure 4. The validation performance plot is provided in Figure 5. Figure 6 shows the regression fit of the ANN model. The values of regression coefficients of training, validation, test, and all data combined are close to 1, which indicates a well-trained model.

### 3.2 Central composite design

The RSM provided the experimental design for the input parameter range is given in Table 2. Table 3 shows the experimental as well as the predicted values of the responses, colour removal efficiency (R1), and electrical energy consumption (R2) for the 30 experimental design runs. From Table 3, it is observed that most of the predicted values are in proximity with the experimental values. However, for the response colour removal efficiency, deviation of the ANN predicted values from the experimental values of Runs 4 and 9 are slightly higher than other runs. While for the response electrical energy consumption, the deviation of the ANN predicted values of Runs 9,13, and 27 seems to be larger. Similarly, it is observed that the RSM predicted and the experimental values of run 27 differs higher than other runs for colour removal efficiency. While Runs 3,17, and 22, the RSM predicted value deviation from the experimental values is larger. The predicted values of Runs 7, 9, 10, 16, 18 differ by minor value from experimental values. The reason for that can be in any predicting model, chances of some data points lying outside the line also knowns as outliers, formed by the regression equation cannot be ruled out. Moreover, it is noteworthy to mention that even after the same inputs the ANN predicts different results for each run.

**Table 3:** Experimental and predicted values for ANN and RSM.

Run No.	Electrolysis Time (minutes) (x_1_)	Current Density (A/m^2^) (x_2_)	pH (x_3_)	Electrolyte concentration g/L (x_4_)	Colour removal efficiency (R1) %			Electrical energy consumption (R2) kWh/kg		
					Exp. values	Predicted values		Exp. values	Predicted values	
						ANN	RSM		ANN	RSM
1	12	60	4	0.5	85.17	85.19	84.33	6.61	5.32	6.24
2	6	100	4	0.5	95.81	96.45	96.13	12.17	11.04	11.78
3	9	80	10	0.4	95	94.12	95.56	14.03	13.16	11.53
4	6	100	8	0.3	96.23	93.43	96.51	13.26	11.76	14.04
5	6	60	4	0.5	78.95	77.23	79.43	5.85	5.15	4.69
6	15	80	6	0.4	99.23	97.92	96.83	26.6	24.86	26.05
7	9	80	6	0.4	98.1	98.56	98.32	16.15	14.91	16.11
8	9	40	6	0.4	87.63	86.03	87.68	9.04	8.32	9.18
9	9	80	6	0.4	98.27	94.67	98.32	16.12	18.34	16.11
10	9	80	6	0.4	98.88	96.99	98.32	16.02	16.29	16.11
11	3	80	6	0.4	86.23	85.25	84.80	5.87	4.18	5.59
12	6	60	8	0.3	87.59	86.12	87.93	8.67	9.21	9.14
13	12	100	8	0.3	93.12	91.39	92.64	26.7	28.93	28.95
14	6	60	8	0.5	90.06	90.52	90.05	8.87	8.32	9.80
15	12	100	4	0.3	96.43	95.34	95.89	34.68	35.15	35.16
16	9	80	6	0.4	98.2	98.06	98.32	16.13	16.62	16.11
17	9	80	6	0.2	92.43	93.18	92.61	27.71	26.87	24.92
18	9	80	6	0.4	98.27	98.12	98.32	16.12	16.17	16.11
19	12	60	8	0.5	94.3	95.36	95.24	16.08	16.01	15.25
20	6	60	4	0.3	76.98	75.43	77.55	10.12	10.09	10.02
21	9	80	6	0.6	98.02	98.33	98.40	14.14	14.19	14.44
22	9	120	6	0.4	99.98	98.51	99.99	30.5	29.24	27.86
23	6	100	4	0.3	92.99	92.67	93.05	18.22	18.53	20.15
24	6	100	8	0.5	99.56	99.11	99.83	11.05	11.09	11.66
25	12	60	4	0.3	93.13	91.49	92.86	17.86	16.88	18.34
26	12	100	4	0.5	99.91	99	99.56	23.4	23.08	24.02
27	15	80	6	0.4	92.43	93.92	96.83	27.71	25.16	26.05
28	12	100	8	0.5	97.68	97.19	96.55	22.3	21.63	23.80
29	12	60	8	0.3	97.41	96.19	96.53	15.58	15.10	17.37
30	9	80	6	0.4	98.2	97.35	98.32	16.13	15.23	16.11

The analysis of variance (ANOVA) generated from the RSM model is shown in Table 4. A p-value of the model or variable is desired to be <0.05. The p-values < 0.005 are considered to be highly significant. For both the responses R1 and R2, the p-value for the models (<0.0001) are less than 0.05 indicates that the models are highly significant (Table 4). For response R1, all the parameters such as electrolysis time (x_1_), current density (x_2_), pH (x_3_), and electrolyte concentration (x_4_) are also highly significant. For response R2 electrolysis time (x_1_), current density (x_2_), and electrolyte concentration (x_4_) are also highly significant.

**Table 4:** The analysis of variance (ANOVA).

Source	Colour removal efficiency, % (R1)			Electrical energy consumption, kWh/kg (R2)		
	df	F-value	p-value	df	F-value	p-value
Model	14	31.91	<0.0001	14	38.59	<0.0001
x₁	1	111.15	<0.0001	1	239.2	<0.0001
x_2_	1	112.82	<0.0001	1	178.49	<0.0001
x_3_	1	26.74	0.0001	1	0.4451	0.5148
x_4_	1	23.06	0.0002	1	56.24	<0.0001
x₁ x_2_	1	71.31	<0.0001	1	15.27	0.0014
x₁ x_3_	1	20.65	0.0004	1	0.0031	0.9565
x₁ x_4_	1	0.1624	0.6927	1	2.63	0.1259
x_2_ x_3_	1	21.96	0.0003	1	9.33	0.008
x_2_ x_4_	1	0.6659	0.4273	1	3.13	0.0971
x_3_ x_4_	1	0.0253	0.8757	1	12.22	0.0033
x₁^2^	1	53.58	<0.0001	1	0.0612	0.8079
x₂^2^	1	13.78	0.0021	1	3.32	0.0885
x₃^2^	1	19.39	0.0005	1	5.63	0.0315
x₄^2^	1	6.09	0.0261	1	7.26	0.0166

Fit Statistics						
R^2^	0.99			0.97		
Adjusted R^2^	0.9964			0.9478		
Predicted R^2^	0.9919			0.8162		

From the F-value, the most significant parameter for R1 is current density, which is followed by electrolysis time, pH, and electrolyte concentration. For response R2 the most influencing parameter is electrolysis time followed by current density, electrolyte concentration, and pH. The coefficient of determination (R^2^) shows the extent of influence of the independent parameters on the response. From the fit statistics in Table 4, the R^2^ values for both responses R1 (>0.98) and R2 (>0.96) are close to 1, which indicates that the independent parameters are largely influencing the response. The predicted R^2^ indicates how well the model predicts unknown data responses, and it should be near to 1 for excellent models. For both the responses, the predicted R^2^ values are close to 1. A close proximity between R2, adjusted R2, and predicted R^2^ is desired and is observed for the model.

**Figure 7:**
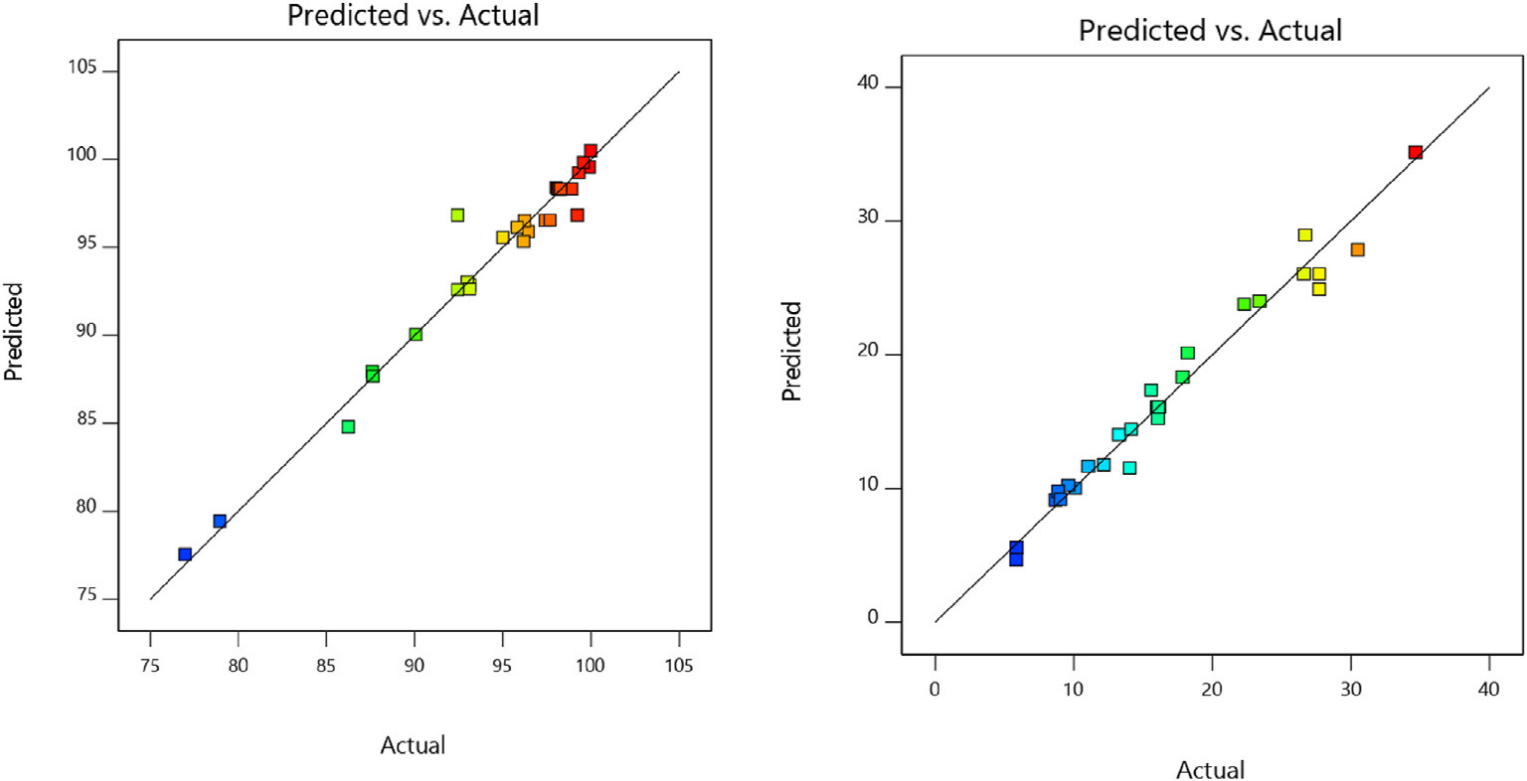
Predicted value Vs actual value plot for (a) Response R1 (b) Response R2.

For prediction, the second order polynomial models were developed for the responses from the RSM. The models are representing the interactive, quadratic, and main impacts of the independent input variables on the response variables. Eq. (9) as well as (10) demonstrate the model for colour removal efficiency (%) and electrical energy consumption, respectively. The models consider only the significant terms (p > 0.05), because the insignificant terms do not affect the process. Figure 7 (a) and (b) show the plot of the predicted values vs. the actual values for response R1 and R2, respectively. For both the responses, the predicted and the actual points are close, indicating ‘good’ fit of models.

(9) Colour removal efficiency (%) = 98.32 + 3.01 x₁+ 3.20 x₂ + 1.84 x₃ - 2.62 x₄ - 3.12 x₁ x₂ - 1.68x₁ x₃ -1.73 x₂ x₃ - 1.88 x₁^2^–1.06 x₂^2^–1.61 x₃^2^–0.7041 x₄^2^

(10) Electrical energy consumption (kWh/kg) = 16.11 + 5.12 x₁+4.67 x₂+1.46 x₄+ 1.67 x₁ x_2_-1.31x₂ x₃ + 1.50 x₃ x₄ + 0.6026x₂^2^–1.01 x₃^2^ + 0.8913 x₄^2^

### 3.3 The impact of operational conditions on the efficiency of colour removal

**Figure 8:**
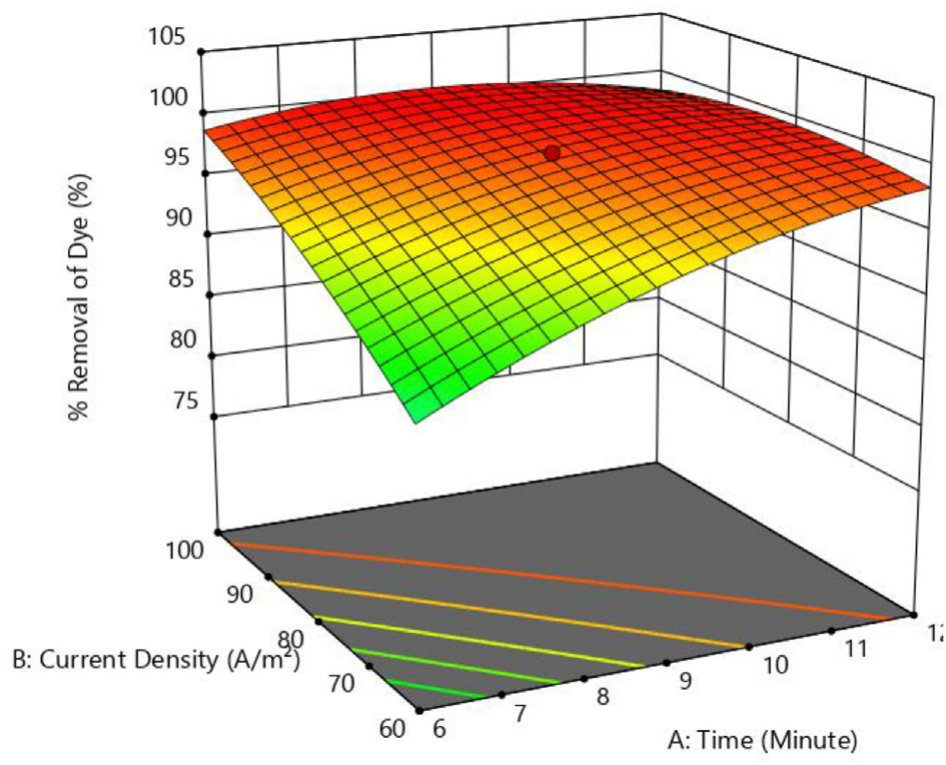
Response surface plots showing the effect of current density Vs stime on colour removal efficiency.

**Figure 9:**
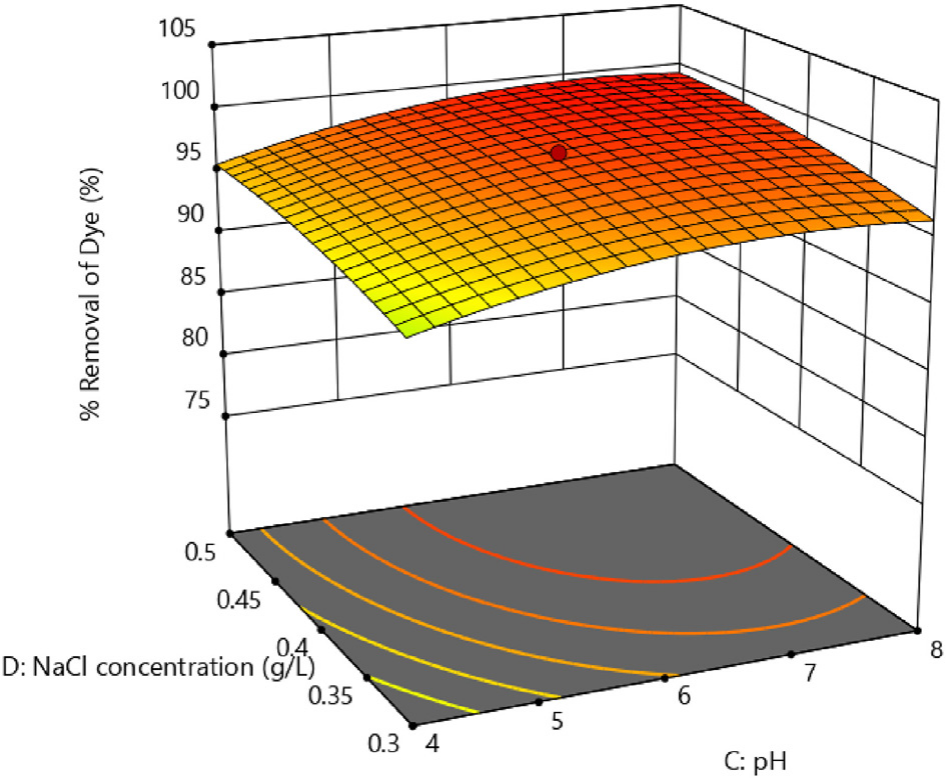
Response surface plot showing the effect of NaCl concentration Vs pH on colour removal efficiency.

Figures 8 and 9 illustrate three-dimensional response surface plots for colour removal rate of brilliant green dye. It shows the impact of four independent parameters on colour removal efficiency by varying two of them while keeping the other two fixed and at the table's central value.

At pH 6, and a NaCl content of 0.4 g/L, Figure 8 depicts the combined influence of time along with current density on colour removal efficiency. The colour removal efficiency increases as time and current density increase. As the reaction time increases, the amount of Fe ions produced at the anode increases. As a result, more Iron hydroxides are produced. This promotes the destabilization of the dye molecules followed by flocculation and precipitation. With the increase in current density, the hydrogen gas generated at the cathode also increases, aiding the coagulation process. Similar results were observed by Verma (2017) [1]. During the electrocoagulation process negatively charged colloidal particles are neutralised by generated metal ions [33]. Increased current density enhances the neutralization of colloidal particles.

**Figure 10:**
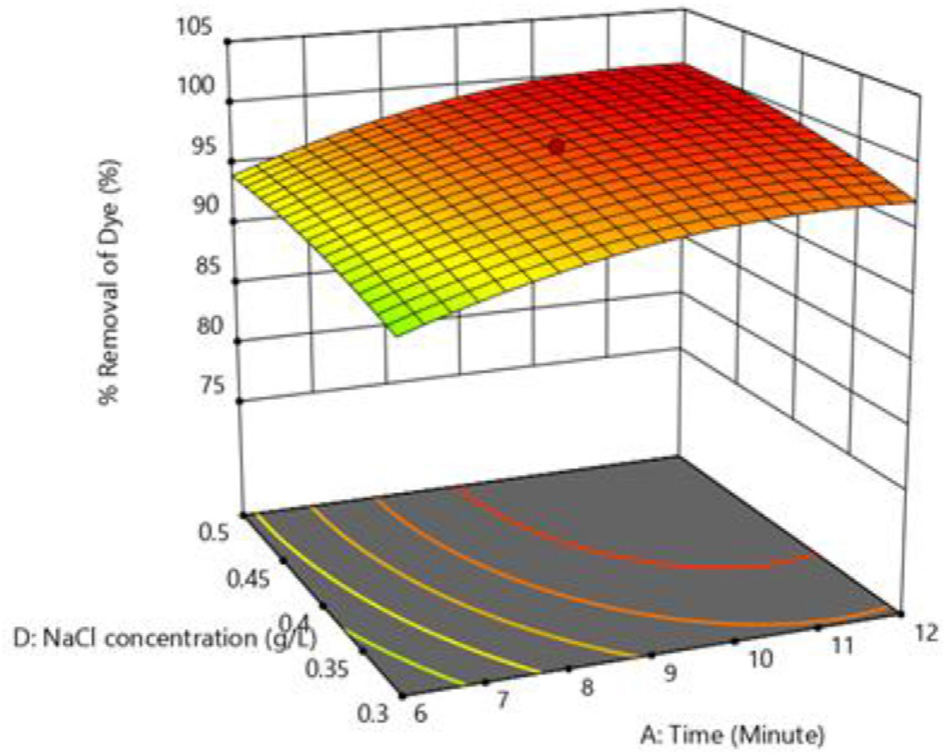
Response surface plot for the effect of NaCl concentration Vs contact time on colour removal efficiency.

**Figure 11:**
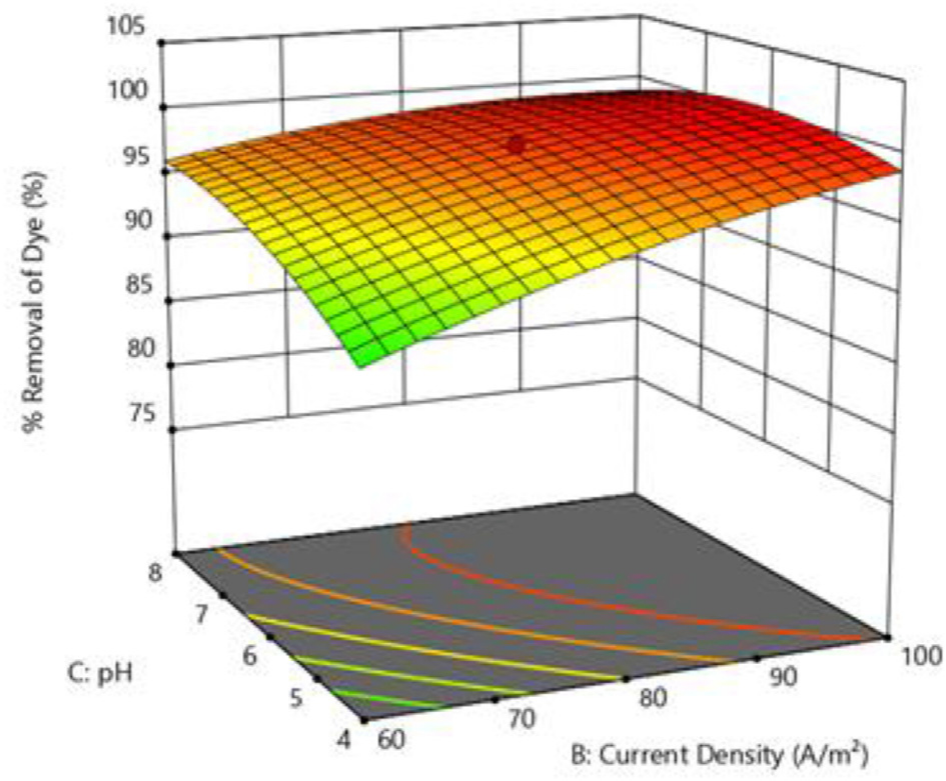
Response surface plot for the effect of pH Vs current density on colour removal efficiency.

Figure 9 depicts the effect of pH and NaCl concentration on colour removal at electrolysis time of 9 min and current density of 80 Am^−2^. From Figure 9, it can be illustrated that the surface plot is comparatively flat. With increasing pH and NaCl concentration, there is a slight increase in colour removal. The pH affects both the ionisation of the dye molecules and the surface characteristics of the adsorbent. BG dye is a cationic and its adsorption is favoured at pH > pH_pzc_ (pH at point of zero charge) [34]. As a result, it is found that increasing the pH from 4 to 8 improves colour removal efficiency. Increase in NaCl (electrolyte) concentration causes increase in the solution conductivity resulting in more concentration of metal ions released from the anode. This might be the apparent cause for the increased efficiency of colour removal with NaCl concentrations. Figure 10 shows the response surface plot for the influence of NaCl concentration against contact time on colour removal efficiency. Figure 11 shows the response surface plot for the influence of pH versus current density on colour removal efficiency.

### 3.4 Effect of operating parameters on electrical energy consumption

**Figure 12:**
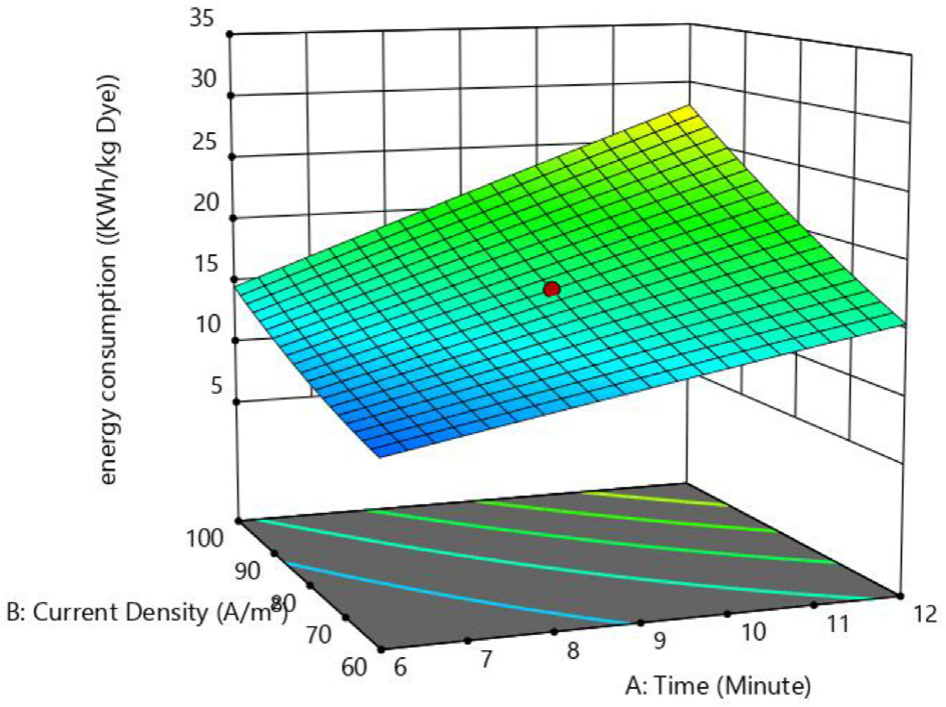
Response surface plots for the effect of current density Vs time on electrical energy consumption.

**Figure 13:**
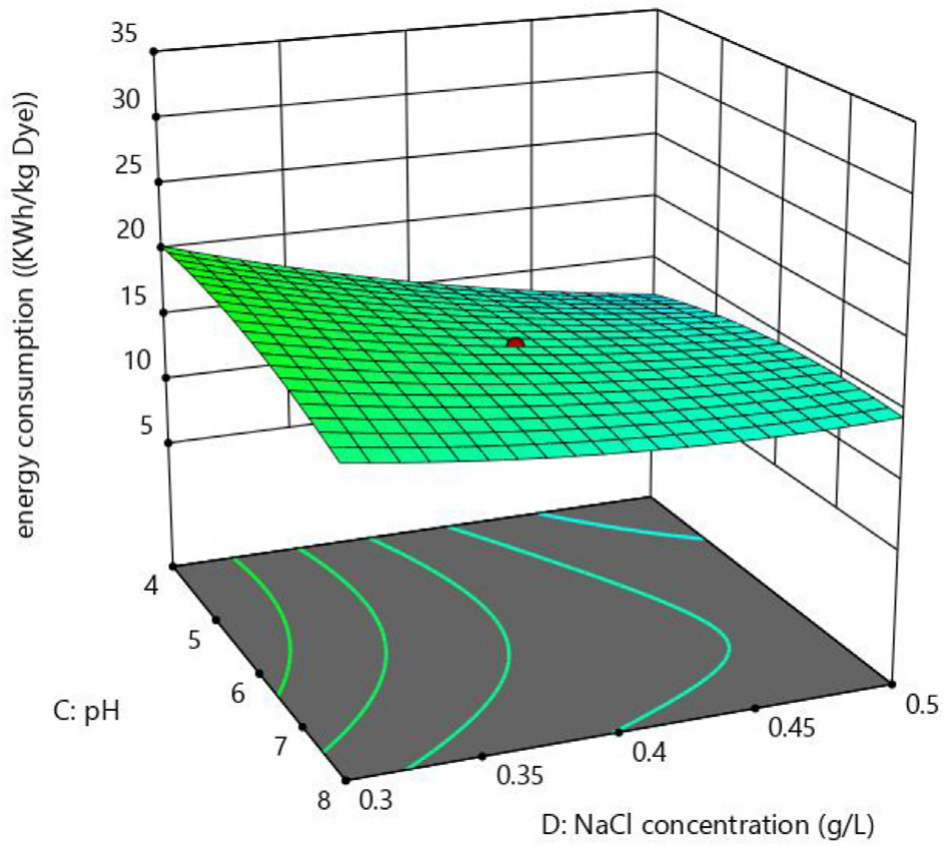
Response surface plots for the effect of NaCl concentration vs pH on electrical energy consumption.

Figures 12 and 13 shows the three-dimensional surface plots for the effect of current density with time and effect of pH and NaCl concentration on electrical energy consumption, respectively electrical energy consumption. The interaction impact of current density combined with electrolysis time on the electrical energy consumption is demonstrated in Figure 12. It is evident that when the current density as well as electrolysis duration rises, the electrical energy consumption increases as well. The reason is the rate of Fe metal ion generation increases with increase in current density and electrolysis time. The effects of pH along with NaCl concentration on energy consumption are shown in Figure 13. It is evident that there is no major effect of pH on electrical energy consumption, whereas the substantial influence of NaCl concentration on electrical energy consumption is found. Electrical energy consumption is decreasing when NaCl concentration is increasing. Similar results were observed by Singh et al, (2016) [35]. At constant current density, a rise in salt concentration lowers cell voltage and reduces power consumption [36].

### 3.5 Optimization of CCD model

**Figure 14:**
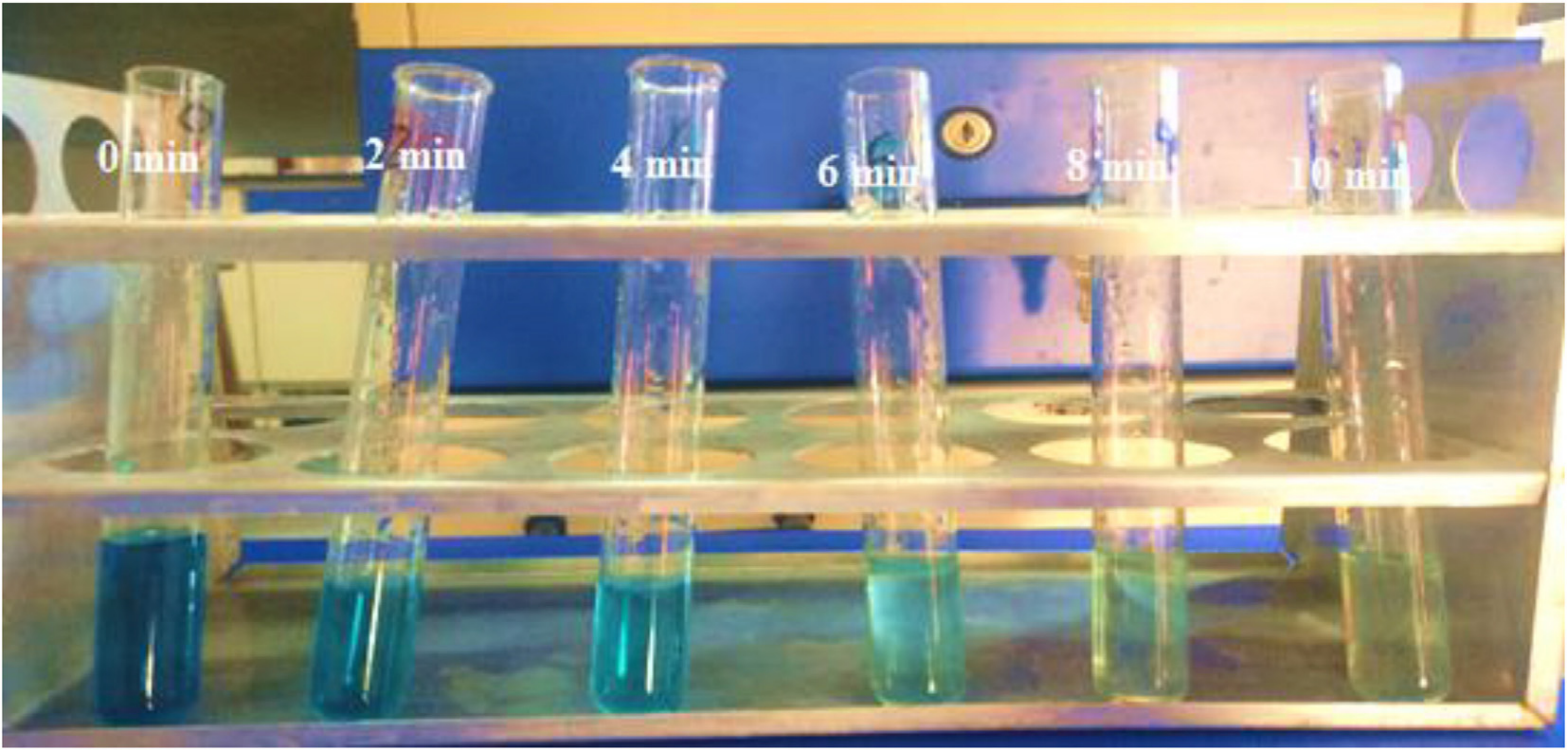
Visual Decolorization at different time intervals at optimum condition.

Experiments were performed under the optimized condition i.e., contact time 10 min, current density 80 A/m^2^, and NaCl concentration 0.5 g/L Table 5 shows the experimental results of two responses, dye removal efficiency, and electrical energy consumption. Predicted values obtained from the RSM are also reported in Table 5. Figure 14 shows the Visual decolorization at different time intervals for optimum condition.

**Table 5:** Optimized condition.

Time (minutes)	Current density (A/m^2^)	pH	NaCl concentration (g/L)	Dye Removal efficiency %		Electrical energy consumption (kWh/kg)	
				Experimental	Predicted	Experimental	Predicted
10	80	5	0.5	98.77	98.83	14.88	14.99

### 3.6 Absorption spectrum under optimum conditions

**Figure 15:**
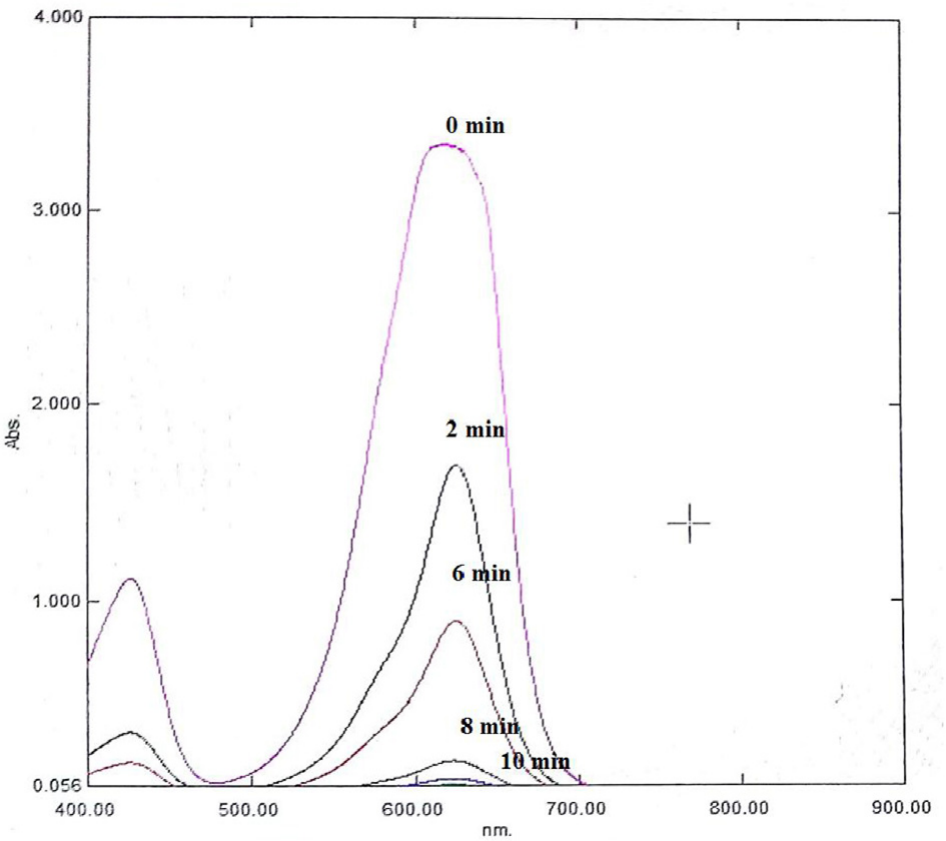
UV-vis absorption spectra obtained at optimum condition.

The UV-vis absorption spectra of the BG dye solution was recorded under optimum conditions for decolorization treatment as shown in Figure 15. The spectra display four bands, one in the visible region (626 nm) due to BG dye and three in the UV-visible region (350, 305, and 255 nm) corresponding to π–π∗ transitions. Continuous decrease in intensity of band at 626 nm from 0 min to 10 min indicates the decolorization.

### 3.7 Kinetic plot under optimum conditions

**Figure 16:**
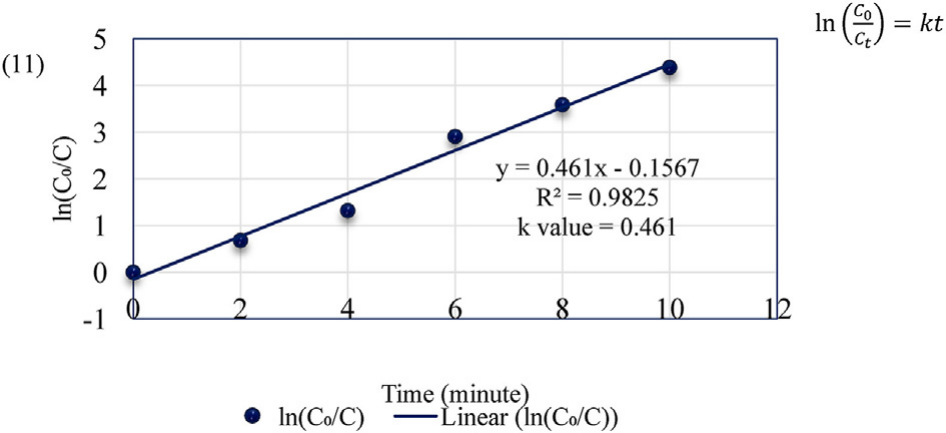
First order kinetic plot for Brilliant Green dye removal process.

A kinetic plot was studied for the optimum condition at constant temperature of 30 °C as shown in Figure 16. According to the first order equation (Eq. 11), the electrocoagulation process followed pseudo first-order reaction kinetics. The reaction rate coefficient, k was observed as shown in Figure 16 where, C_0_ and C_t_ are the initial and final dye concentration (mg/L) of BG dye before and after time t (minutes), k is the reaction rate coefficient, and m is the order of reaction.

(11) $$\ln\left(\frac{C_{0}}{C_{t}}\right)=kt$$

## 4 Conclusion

The electro coagulation technique was examined for the decolourization of BG dye solution in this research. A process was influenced by parameters such as current density, initial pH, electrolysis time, and the electrolyte (NaCl) concentration. An ANN was used to model and train the process for prediction. The RSM approach was applied to optimize the procedure, which reduce the time and experimental work. The process was effectively modelled using a second order polynomial CCD design, giving prediction models for two response variables: dye removal efficiency and electrical energy consumption. For the decolorization of BG dye solution, the maximum dye removal efficiency (>98%) and minimal electrical energy consumption (15 kWh/kg) were achieved under optimum conditions. Overall, the procedure was determined to be successful in removing the BG dye.

## Declarations

### Author contribution statement

Manisha S. Kothari & Kinjal G. Vegad: Performed the experiments; Analyzed and interpreted the data; Wrote the paper.

Kosha A. Shah: Conceived and designed the experiments; Analyzed and interpreted the data; Contributed reagents, materials, analysis tools or data; Wrote the paper.

Ashraf Aly Hassan: Conceived and designed the experiments; Analyzed and interpreted the data; Contributed reagents, materials, analysis tools or data.

### Funding statement

This research did not receive any specific grant from funding agencies in the public, commercial, or not-for-profit sectors.

### Data availability statement

Data will be made available on request.

### Declaration of interests statement

The authors declare no conflict of interest.

### Additional information

No additional information is available for this paper.
